# Time Course of Lipid Peroxidation, Antioxidant Markers and C-Reactive Protein Following Simulated Soccer Match-Play: A 72-h Study in Semi-Professional Players

**DOI:** 10.3390/jcm15218394

**Published:** 2026-10-28

**Authors:** Mustapha Bouchiba, Mouna Turki, Safaa M. Elkholi, Omar Trabelsi, Mohamed Amine Grati, Mohamed Amine Bouzid

**Affiliations:** 1 Laboratoire Hypoxie & Poumon, UMR INSERM U1272, Université Sorbonne Paris Nord, 93017 Bobigny, France; 2 Laboratory of Biochemistry, “Molecular Basis of Human Diseases”, LR19ES13, Faculty of Medicine of Sfax, University of Sfax, Sfax 3029, Tunisia; 3 Department of Rehabilitation Sciences, College of Health and Rehabilitation Sciences, Princess Nourah bint Abdulrahman University, P.O. Box 84428, Riyadh 11671, Saudi Arabia; 4 Research Unit: Physical Activity, Sport and Health, UR18JS01, National Observatory of Sport, Tunis 1003, Tunisia; 5 High Institute of Sports and Physical Education of Kef, University of Jendouba, Le Kef 7100, Tunisia; 6 Faculty of Medicine of Sfax, University of Sfax, Sfax 3029, Tunisia; 7 Research Laboratory, Education, Motricité, Sport et Santé (EM2S), LR19JS01, High Institute of Sport and Physical Education, University of Sfax, Sfax 3000, Tunisia

**Keywords:** exercise recovery, malondialdehyde, conjugated dienes, antioxidant defense, football

## Abstract

***Background/Objectives*:** Soccer match-play triggers muscle damage and a marked increase in reactive oxygen species production, but early and late lipid peroxidation products have rarely been tracked together with non-enzymatic antioxidants and C-reactive protein in the same players over a full 72-h window. This study characterized the recovery kinetics of oxidative damage markers (conjugated dienes, malondialdehyde) and antioxidant/defense markers (uric acid, bilirubin, reduced glutathione, gamma-glutamyl transferase), together with C-reactive protein, and explored the relationships between these markers’ recovery responses. ***Methods*:** Twelve semi-professional male soccer players completed the Loughborough Intermittent Shuttle Test (LIST). Venous blood samples were collected at Baseline, 0 h, 24 h, 48 h, and 72 h post-LIST. Repeated-measures ANOVA or the Friedman test was applied depending on normality, with Holm-adjusted pairwise comparisons against Baseline and exploratory Spearman correlations. ***Results*:** Uric acid and reduced glutathione peaked at 0 h and no longer differed significantly from baseline from 24 h and 48 h onward, respectively. CRP increased significantly at 24 h, whereas bilirubin showed only a non-significant 24-h peak. Conjugated dienes remained significantly elevated at every time point up to 72 h, whereas malondialdehyde differed significantly from baseline only at 0 h. In exploratory analyses not corrected for multiple testing, uric acid correlated with bilirubin and, on an area-under-the-curve basis, with C-reactive protein, and conjugated dienes correlated with malondialdehyde. ***Conclusions*:** The uric acid and reduced glutathione responses and the C-reactive protein response resolved within 24 to 48 h, whereas conjugated dienes remained elevated up to 72 h. These findings indicate prolonged alterations in selected circulating biomarkers rather than direct evidence of persistent muscle oxidative damage or incomplete functional recovery, and their relevance to congested fixture scheduling warrants investigation alongside performance outcomes.

## 1. Introduction

Soccer is a high-intensity intermittent sport characterized by repeated sprints, accelerations, decelerations, and rapid directional changes interspersed with lower-intensity activity [1]. During a 90-min match, elite male players typically cover approximately 10–12 km, with a substantial proportion performed at high intensity depending on playing position and the thresholds used to classify running speed [1]. These demands impose considerable metabolic, mechanical, and neuromuscular stress, including muscle glycogen depletion, reduced sprint capacity, and transient impairment of contractile function [2,3]. Recovery may remain incomplete for 48–72 h, which is particularly relevant during congested schedules when players may be required to compete twice within the same week [3,4].

Perturbations in redox homeostasis may contribute to post-exercise muscle dysfunction and delayed recovery. Oxidative stress has traditionally been described as an imbalance favoring oxidants over antioxidant defenses, with the potential to disrupt cellular function and promote oxidative damage [5]. However, reactive oxygen and nitrogen species are not exclusively detrimental, as transient increases contribute to redox-sensitive signaling and exercise adaptation [6]. When reactive species formation becomes excessive or inadequately controlled, oxidative modification of lipids, proteins, and other cellular components may impair muscle function [5,7]. Experimental evidence also indicates that reactive species contribute to contraction-induced fatigue and may alter sarcoplasmic reticulum Ca^2+^ handling through redox-sensitive modification of the ryanodine receptor [7,8]. Thus, both the magnitude and duration of post-exercise redox disturbances may influence recovery following demanding intermittent exercise.

Soccer match-play has been shown to induce marked but biomarker-specific changes in oxidative and antioxidant status during recovery [9,10]. Ascensão et al. [9] reported alterations in lipid peroxidation and antioxidant-related variables throughout a 72-h post-match period. Ispirlidis et al. [11] demonstrated distinct temporal profiles, with C-reactive protein (CRP) and thiobarbituric acid-reactive substances reaching their highest values at approximately 24 h, protein carbonyls at approximately 48 h, and uric acid at approximately 72 h. Fatouros et al. [12] similarly observed prolonged changes in lipid and protein oxidation, glutathione status, uric acid, and total antioxidant capacity over 72 h of recovery. Silva et al. [10] also reported post-match alterations in malondialdehyde (MDA), antioxidant-related variables, uric acid, and CRP in professional players. These findings indicate that post-match redox recovery is dynamic and cannot be adequately characterized using a single biomarker or a single post-exercise time point.

Competitive matches provide high ecological validity but introduce substantial variability in physical load, playing time, tactical demands, environmental conditions, and match context [1,9,10]. Standardized soccer-simulation protocols provide an experimental alternative by allowing the exercise stimulus to be reproduced under controlled conditions. The Loughborough Intermittent Shuttle Test (LIST) was developed to reproduce the intermittent activity pattern characteristic of soccer and provides a reproducible soccer-specific exercise stimulus [13]. However, simulated match-play should not be considered physiologically identical to competitive soccer. In a direct comparison, Magalhães et al. [14] showed that actual match-play produced greater disturbances than the LIST in several redox variables, including MDA, uric acid, protein sulfhydryls, and total antioxidant status. Nevertheless, the standardized nature of the LIST reduces between-match variability and provides a useful model for examining the temporal course of recovery [13,14]. Compared with competitive match-play, the multi-marker redox response during prolonged recovery from standardized simulated soccer remains less well characterized.

A multi-marker approach may therefore provide a more complete assessment of post-exercise redox recovery. MDA and conjugated dienes provide complementary indices of lipid peroxidation [12,15], whereas reduced glutathione (GSH) represents an important component of cellular antioxidant defense [5,12]. Uric acid contributes to circulating antioxidant capacity and has been shown experimentally to attenuate exercise-induced oxidative stress [16], while bilirubin possesses established antioxidant properties and may protect against lipid oxidation [17]. Gamma-glutamyl transferase (GGT) is involved in extracellular glutathione metabolism and may provide complementary information on glutathione-related redox processes, although it is better regarded as an indirect redox-related marker than as a classical antioxidant enzyme [18]. In parallel, CRP provides an index of the systemic acute-phase inflammatory response and may help determine whether inflammation follows a similar or distinct recovery trajectory from redox-related biomarkers [10,11].

Although several studies have described oxidative stress and inflammatory responses during the 72 h following competitive soccer matches [9,10,11,12], the present study was designed to extend this literature in three respects. First, it combines two lipid peroxidation products that reflect different stages of the peroxidation process, conjugated dienes as early products and MDA as a later end-product [15], so that their time courses can be compared within the same players; to our knowledge, conjugated dienes have rarely been tracked during post-match recovery in soccer. Second, it measures these lipid peroxidation products together with non-enzymatic antioxidants (uric acid, bilirubin, and GSH), a glutathione-related enzyme (GGT), and CRP in the same participants and at the same sampling times, so that the relative timing of the redox-related and acute-phase responses can be compared directly rather than across studies that used different exercise stimuli, populations, and assays. Third, it uses a standardized soccer simulation, which limits the between-match variability in external load that complicates the interpretation of time courses after competitive matches [1,14]. In addition, individual-level analyses based on peak changes and areas under the curve allow an exploratory examination of whether the magnitude of the response of one marker tracks with that of another, which may help generate hypotheses for future studies.

Therefore, the aim of this study was to characterize the 72-h recovery kinetics of redox-related biomarkers following simulated soccer match-play and to examine the accompanying systemic inflammatory response using C-reactive protein. We hypothesized that the measured biomarkers would exhibit distinct temporal responses across the 72-h recovery period.

## 2. Materials and Methods

### 2.1. Participants

Twelve semi-professional male soccer players (23.2 ± 0.7 years) volunteered to participate in this study (see Table 1). All players underwent a medical screening prior to inclusion to confirm the absence of chronic disease and their fitness for high-level sporting activity, and none reported a history of motor or neurological disorders or chronic use of anti-inflammatory or analgesic medication. Participants were instructed to abstain from any pain-relief or anti-inflammatory medication, nutritional supplementation, and unaccustomed strenuous exercise for the 7 days preceding the trial and throughout the experimental period. Players competed in the third division of the Tunisian soccer league and had at least three years of competitive soccer experience. During the preceding competitive season, they typically completed 3–4 training sessions per week in addition to one official match per week. The study was conducted during the transition from the off-season to the pre-season, when participants were not engaged in structured team training or competitive matches.

The study was approved by the local Clinical Research Ethics Committee (CPP Sud, reference CPP SUD N° 0104/2018) and conducted in accordance with the Declaration of Helsinki 2013. All participants provided written informed consent after receiving a detailed explanation of the experimental procedures and associated risks.

### 2.2. Study Design and Simulated Match Protocol

Participants attended the laboratory for two familiarization visits before the experimental trial to become accustomed to the testing procedures and to the demands of the simulated soccer match-play. Anthropometric data (age, body mass, height) were recorded, and maximal oxygen uptake (VO_2_max, mL·kg^−1^·min^−1^) was estimated from the multistage 20 m shuttle run test [19] to individualize the running speeds used in the match protocol.

**Figure 1:**
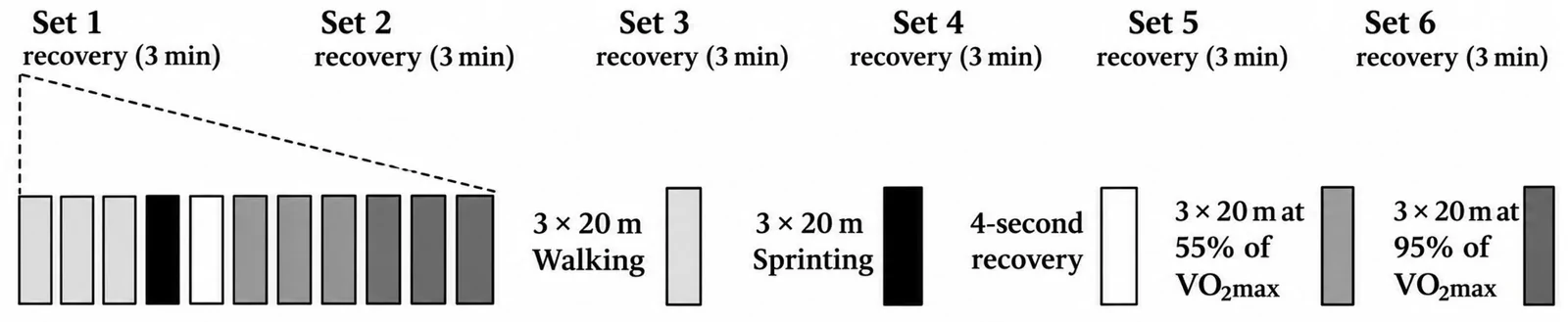
Schematic representation of the Loughborough Intermittent Shuttle Test (LIST) protocol.

On the day of the experimental trial, participants completed the Loughborough Intermittent Shuttle Test (LIST), a validated field protocol that reproduces the activity pattern of a soccer match [13]. The LIST consisted of six 15-min exercise blocks separated by 3-min passive recovery periods. Each block required participants to complete repeated cycles of 3 × 20 m at walking pace, 1 × 20 m at maximal sprint speed followed by a 4-s recovery, 3 × 20 m at a speed corresponding to 55% of individual estimated VO_2_max, and 3 × 20 m at 95% of individual estimated VO_2_max. The structure of the LIST protocol is illustrated in Figure 1. As in the original protocol [13], the walking, jogging (55% of estimated VO_2_max), and running (95% of estimated VO_2_max) speeds were dictated by pre-recorded audio signals. The audio tracks were individualized using Audacity software version 2.2.2, with signal intervals adjusted to match each participant’s prescribed speeds throughout the six blocks. All twelve participants completed the six exercise blocks of the protocol.

**Figure 2:**
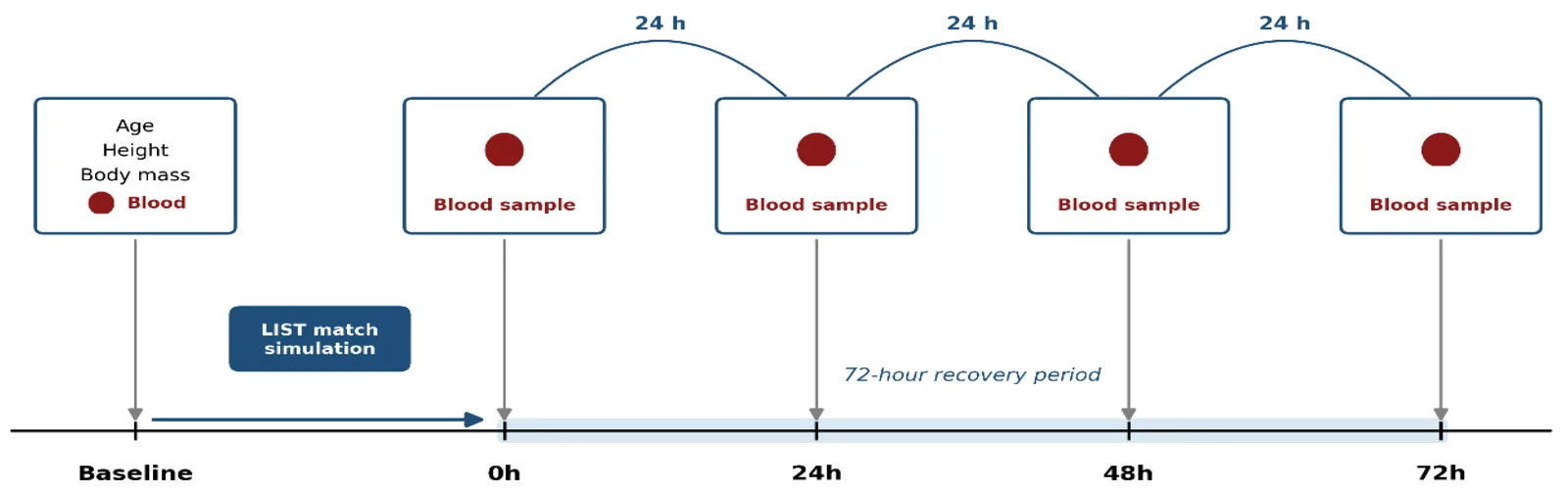
Schematic representation of the overall experimental procedure. **Note.** Anthropometric data (age, height, body mass) were recorded at Baseline before the Loughborough Intermittent Shuttle Test (LIST) match simulation. Venous blood samples were drawn at Baseline and at 0 h, 24 h, 48 h, and 72 h post-LIST, spanning the full 72-h recovery period.

Venous blood samples were collected at five time points: immediately before the LIST (Baseline), immediately after its completion (0 h), and after 24, 48, and 72 h of recovery. No recovery intervention (e.g., cold-water immersion, massage, compression garments, or pharmacological or nutritional supplementation) was administered between the match and any of the follow-up assessments, so that the recovery kinetics observed reflect the natural, unassisted time course of the measured markers. Participants avoided caffeine and alcohol for at least 24 h and strenuous exercise for at least 48 h before the trial and maintained their habitual sleep schedule throughout the study period. The overall timeline of the experimental procedure is illustrated in Figure 2.

Throughout the 72-h recovery period, participants were instructed to maintain their habitual diet and fluid intake, to refrain from caffeine, alcohol, and nutritional supplements, and to avoid any strenuous or unaccustomed physical activity. Dietary intake, hydration status, and daily physical activity were not monitored; this is acknowledged as a limitation (Section 4.1). Baseline and follow-up blood samples on the three recovery days were collected at approximately 9:00 a.m. before breakfast to minimize potential circadian variation. The baseline sample was obtained after an overnight fast and was followed by a standardized breakfast before the LIST. The immediate post-LIST sample was collected upon completion of the LIST protocol.

### 2.3. Blood Sampling and Biochemical Analyses

After 5 min of seated rest, an 8 mL venous blood sample was drawn from an antecubital forearm vein at each time point and distributed into lithium-heparin (4 mL) and K_2_EDTA (3.5 mL) tubes. Samples were transported immediately to the biochemistry laboratory, centrifuged at 3000 rpm for 15 min, and the resulting plasma was aliquoted and stored at −80 °C until analysis.

Uric acid, plasma levels of total bilirubin, gamma-glutamyl transferase, and C-reactive protein were determined from the lithium-heparin tube using enzymatic and immunoturbidimetric methods on an automated clinical chemistry analyzer, following the manufacturer’s validated protocols.

Malondialdehyde, reduced glutathione, and conjugated dienes were determined from the K_2_EDTA tube.

#### 2.3.1. Determination of Lipid Peroxidation (MDA)

Plasma MDA was determined using a thiobarbituric acid-based spectrophotometric method adapted from Wong et al. [20]. A 0.5-mL plasma aliquot was mixed with 0.1 mL of Tris-HCl buffer (pH 7.2) and incubated at 95 °C. Subsequently, 0.5 mL of the reaction mixture was combined with 9 mL of distilled water and 2 mL of 0.6% thiobarbituric acid. The mixture was heated in a boiling water bath for 30 min and then allowed to cool.

Following cooling, 5 mL of *n*-butanol was added and the samples were vigorously mixed. The n-butanol layer was separated by centrifugation at 1000 *g* for 10 min, and the production amount of malonic MDA was measured at 532 nm in a spectrophotometer (Libra S21, Biochrom, Holliston, MA, USA).

#### 2.3.2. Measurement of GSH Levels

Reduced glutathione (GSH) was determined colorimetrically using Ellman’s reagent, 5,5′-dithiobis-(2-nitrobenzoic acid) (DTNB) [21]. The assay is based on the reaction of GSH with DTNB, producing thionitrobenzoic acid (TNB), which is quantified at 412 nm. A 4% aqueous sulfosalicylic acid solution and a Tris-EDTA buffer containing 0.25 M Tris base and 0.02 M EDTA were used. The buffer was prepared by mixing equal volumes of 0.5 M Tris base (3 g in 50 mL distilled water) and 0.04 M EDTA (0.58 g in 50 mL distilled water), with the final pH adjusted to 8.2. A fresh 10 mM DTNB solution was prepared in methanol and protected from light. A GSH calibration curve ranging from 0.03 to 1 mmol/L was used. GSH concentrations were determined according to Jollow et al. [22], initially expressed as nmol/mL plasma and subsequently normalized to protein concentration as nmol GSH/mg protein.

The calculation was performed as:

$$\mathrm{GSH}\;(\mathrm{nmol}/\mathrm{mg})\;=\frac{\left(DO-b/a\times FD\times 1000\right)}{\mathrm{protein}\;\mathrm{concentration}\;\left(\mathrm{mg}/\mathrm{mL}\right)}$$

where ‘DO’ is the optical density, ‘a’ is the slope of the GSH calibration curve (μmol/mL), ‘b’ is the y-intercept, *FD* is the dilution factor, and 1000 is the conversion factor from μmol to nmol.

#### 2.3.3. Conjugated Dienes Assay

Conjugated dienes, representing primary products of lipid oxidation characterized by conjugated double bonds, were quantified by UV spectrophotometry [23]. Plasma lipids were extracted using a chloroform–methanol mixture. Briefly, 25 µL of plasma was added to 3 mL of chloroform/methanol (2:1, *v*/*v*) in a 15-mL conical tube without agitation, and the mixture was centrifuged at 3000 rpm for 5 min. Subsequently, 2 mL of the supernatant was transferred to a glass tube and incubated overnight at 45 °C to allow solvent evaporation and formation of a lipid extract on the tube wall. The dried extract was then reconstituted in 1 mL of methanol using a vortex mixer, and optical density was measured at 233 nm. Results were expressed as optical density units (OD units).

#### 2.3.4. Routine Biochemistry Parameters

Bilirubin (BIL), uric acid (UA), gamma-glutamyl transferase (GGT), and C-reactive protein (CRP) were analyzed using a Cobas 6000^®^ automated biochemistry analyzer (c501 module; Roche Diagnostics, Mannheim, Germany). TBIL was determined using the diazo reaction [24], while UA was measured enzymatically at 550 nm [25]. GGT was assessed using an enzymatic colorimetric method with L-γ-glutamyl carboxy-3-nitro-4-anilide as the substrate, in accordance with IFCC recommendations [26]. CRP concentrations were determined using an immunoturbidimetric assay [27].

### 2.4. Statistical Analysis

Data were analyzed using Python (SciPy and Pingouin libraries). Descriptive statistics are reported as mean and standard deviation (SD) for normally distributed variables and as median and interquartile range (IQR) for non-normally distributed variables. The normality of the distribution of each biomarker at each time point (Baseline, 0 h, 24 h, 48 h, and 72 h) was assessed with the Shapiro–Wilk test.

For biomarkers meeting the normality assumption at all time points (uric acid, gamma-glutamyl transferase, and reduced glutathione), a one-way repeated-measures analysis of variance (ANOVA) was used to test the effect of time. Mauchly’s test was used to assess sphericity, and the Greenhouse–Geisser correction was applied to the degrees of freedom when sphericity was violated (*p* < 0.05). Effect sizes for the omnibus test are reported as partial eta-squared.

For biomarkers that violated the normality assumption at one or more time points (bilirubin, C-reactive protein, conjugated dienes, and malondialdehyde), the non-parametric Friedman test was used, with Kendall’s *W* reported as the corresponding effect size.

When the omnibus test was significant, planned pairwise comparisons of each post-LIST time point against the Baseline value were performed using paired-samples *t* tests (for the parametric markers, with Cohen’s *dz* as the effect size) or Wilcoxon signed-rank tests (for the non-parametric markers, with the matched rank-biserial effect size *r*). *p* values from pairwise comparisons were adjusted for multiple comparisons within each marker using the Holm–Bonferroni method. Statistical significance was set at *p* < 0.05 for all tests.

To explore whether the recovery response of one marker tracked with that of another, exploratory Spearman correlations were computed across participants using each marker’s peak change from Baseline, defined as the largest post-LIST value minus the Baseline value. Correlations were also computed using the area under the curve (AUC) of each marker across the post-LIST time points (0 h, 24 h, 48 h, and 72 h; trapezoidal rule) to capture total exposure over the recovery period. Because these correlations involved multiple pairwise comparisons in a small sample (*N* = 12), they were treated as exploratory and hypothesis-generating, and no correction for multiple comparisons was applied to this set of tests. All 21 possible pairs of the seven markers were examined on each basis (42 tests in total), so that about two nominally significant correlations would be expected by chance alone at α = 0.05 even in the absence of any true association; for this reason, the correlations are reported descriptively and are not used to support mechanistic conclusions. To convey their precision, 95% confidence intervals (CIs) were computed for each reported coefficient using Fisher’s *z* transformation.

No a priori sample size calculation was performed. The sample size was determined pragmatically by the number of eligible players who volunteered and were available for all laboratory visits and follow-up samplings during the transition period. A sensitivity power analysis was therefore performed in Python version 3.14.6 (SciPy; noncentral *t* and *F* distributions) to indicate the smallest effects that this sample could reliably detect (two-tailed α = 0.05, power = 0.80). With *N* = 12, the study could detect within-participant effects of *dz* ≥ 0.89 for a single paired comparison (*dz* ≥ 1.13 at the most stringent Holm-adjusted threshold, α = 0.0125), an omnibus effect of *f* ≥ 0.33 (partial eta-squared ≥ 0.10) for the five-level repeated-measures ANOVA (assuming a correlation of 0.5 among repeated measures and sphericity), and Spearman correlations of 0.73 or greater in absolute value. Smaller effects could not be detected reliably, and non-significant results should be interpreted accordingly.

## 3. Results

Twelve semi-professional players completed the simulated soccer match and provided complete blood samples at all five time points (Baseline, 0 h, 24 h, 48 h, and 72 h post-LIST). Descriptive statistics for all oxidative damage and antioxidant/defense markers across the recovery period are presented in Table 2.

### 3.1. Markers Analyzed with Repeated-Measures ANOVA

Uric acid changed significantly over time (Table 2). Mean concentration increased by approximately 20% immediately after the LIST (0 h; *dz* = 1.32) and no longer differed significantly from Baseline from 24 h onward, although mean values remained above Baseline at 24 h (+13%) and 48 h (+7%) and returned to Baseline values only at 72 h (Table 3).

Gamma-glutamyl transferase (GGT) did not change significantly over time (Table 2). Because the omnibus test was not significant, no pairwise comparisons were performed for this marker, in accordance with the predefined analysis plan (Section 2.4).

Reduced glutathione (GSH) changed significantly over time (Table 2), with mean values approximately 45% above Baseline at 0 h (*dz* = 1.28) and 37% above Baseline at 24 h (*dz* = 0.88); differences from Baseline were no longer significant at 48 h and 72 h (Table 3).

### 3.2. Markers Analyzed with the Friedman Test

Bilirubin showed a significant omnibus effect of time (Table 2), with its highest median value at 24 h (+57% relative to Baseline), but no individual comparison against Baseline remained significant after Holm adjustment (Table 3).

C-reactive protein changed significantly over time (Table 2). Its median value at 24 h was 2.5-fold higher than at Baseline, and this was the only time point that differed significantly from Baseline (Table 3).

Conjugated dienes showed the largest effect of time among the markers analyzed with the Friedman test (Kendall’s *W* = 0.622; Table 2). Median values were 1.7-fold higher than Baseline at 0 h, approximately fourfold higher at 24 h and 48 h, and still 2.7-fold higher at 72 h, and all post-LIST time points differed significantly from Baseline (Table 3).

Malondialdehyde (MDA) changed significantly over time (Table 2), with a median value approximately 52% above Baseline at 0 h, the only time point that differed significantly from Baseline after Holm adjustment (Table 3).

**Figure 3:**
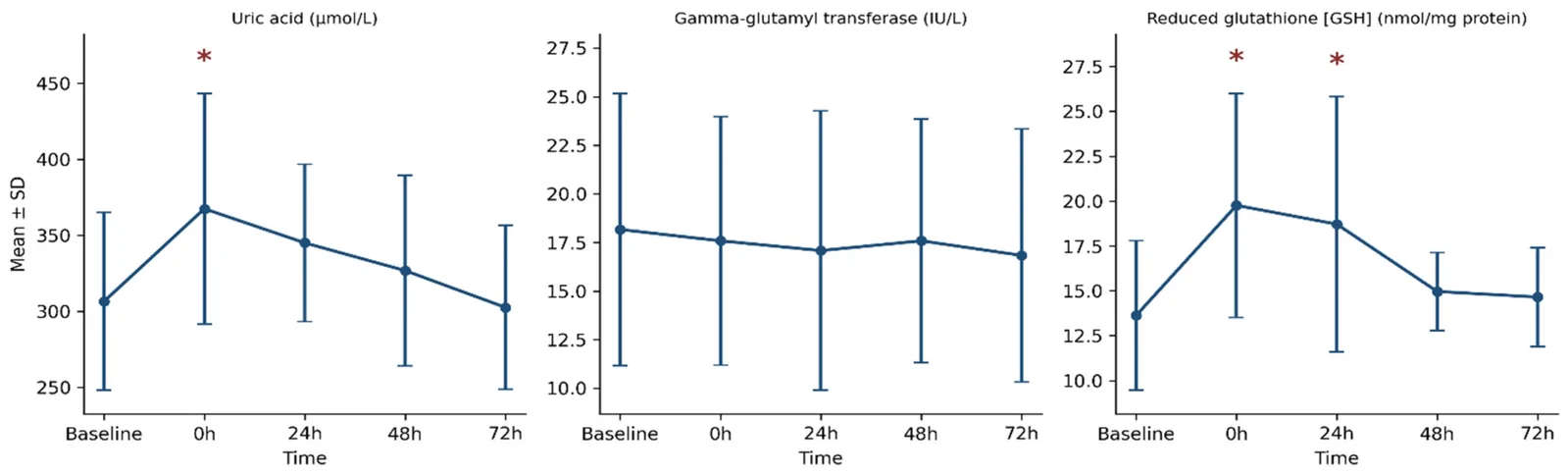
Recovery kinetics (mean ± SD) of uric acid, gamma-glutamyl transferase, and reduced glutathione (GSH) from Baseline to 72 h following simulated soccer match-play. **Note.** These markers were analyzed with repeated-measures ANOVA. Asterisks (∗) denote Holm-adjusted *p* < 0.05 vs. Baseline.

**Figure 4:**
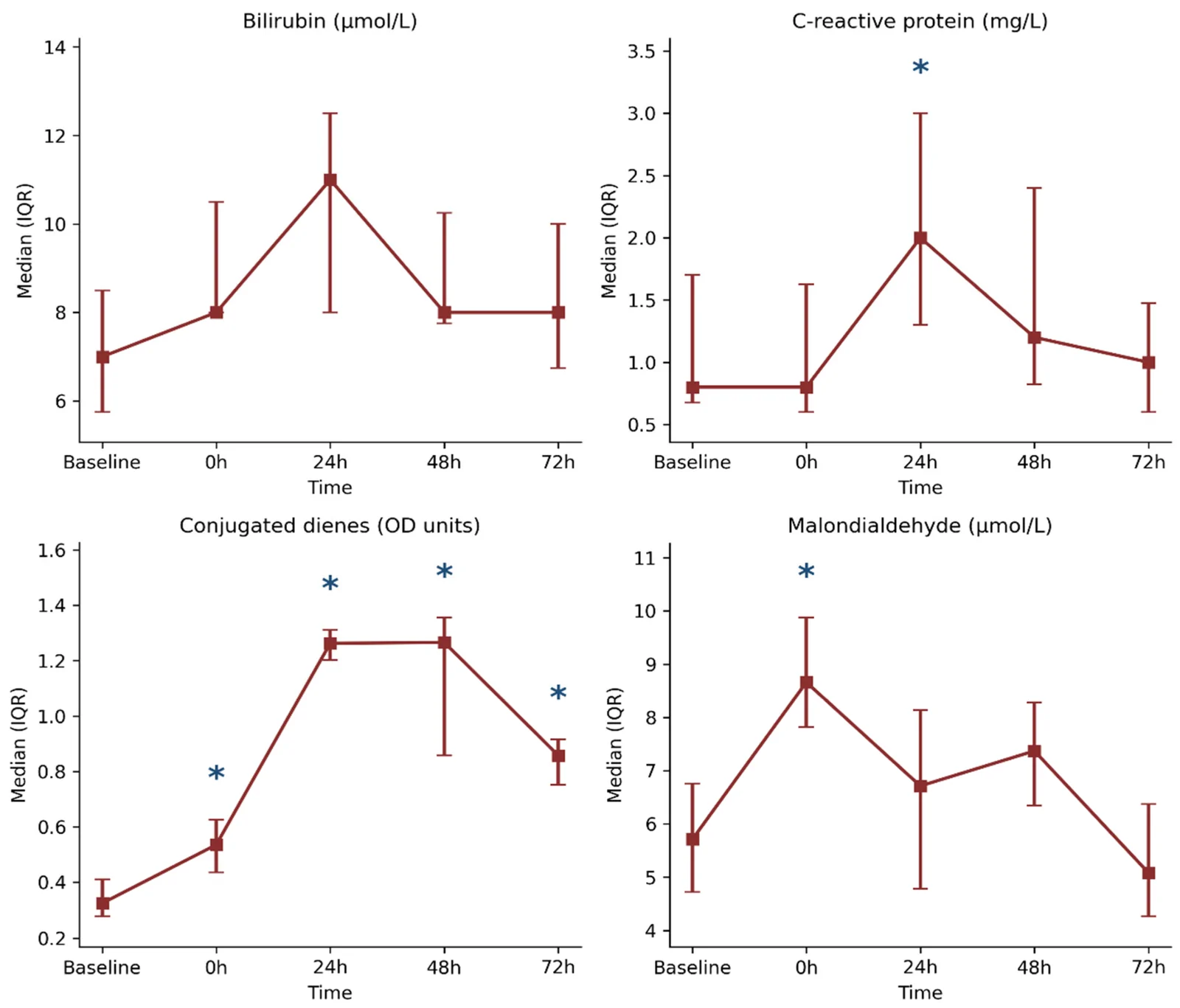
Recovery kinetics (median [IQR]) of bilirubin, C-reactive protein, conjugated dienes, and malondialdehyde (MDA) from Baseline to 72 h following simulated soccer match-play. **Note.** These markers were analyzed with the Friedman test. Asterisks (∗) denote Holm-adjusted *p* < 0.05 vs. Baseline.

Overall, the pattern of change across the recovery period differed by marker. Uric acid, GSH, and MDA reached their highest values immediately after the LIST (0 h) and no longer differed significantly from Baseline from 24 h (uric acid and MDA) or 48 h (GSH) onward. Bilirubin and C-reactive protein reached their highest values later, at 24 h, although only C-reactive protein differed significantly from Baseline at that time point. Conjugated dienes rose progressively to reach their highest values at 24 h to 48 h and remained significantly above Baseline at 72 h, whereas GGT did not change significantly. The overall recovery kinetics of the seven markers across the five time points are illustrated in Figure 3 and Figure 4.

### 3.3. Exploratory Correlation Analysis

Exploratory Spearman correlations yielded three nominally significant relationships (see Table 4): two based on peak changes and one based on the area under the curve (AUC). The peak changes in the two lipid peroxidation markers, conjugated dienes and malondialdehyde, were positively correlated (*r* = 0.60, *p* = 0.039; 95% CI 0.04 to 0.87), as were those of uric acid and bilirubin (*r* = 0.65, *p* = 0.023; 95% CI 0.12 to 0.89). When correlations were instead computed using the area under the curve across the post-LIST period, uric acid and C-reactive protein were also positively correlated (*r* = 0.71, *p* = 0.009; 95% CI 0.23 to 0.91). None of these correlations would remain significant after correction for the number of tests performed, and their wide confidence intervals indicate that the true associations could range from negligible to very strong.

## 4. Discussion

This study characterized the combined 72-h time course of early (conjugated dienes) and late (MDA) lipid peroxidation products, non-enzymatic antioxidants, GGT, and C-reactive protein, measured in the same players after a standardized simulated soccer match. Consistent with previous work using the LIST protocol and competitive match-play [9,10,14], our findings show that simulated soccer match-play is sufficient to alter circulating markers of both lipid peroxidation and antioxidant defense, and that the time required for these circulating markers to return to Baseline differs substantially between markers. Because only plasma biomarkers were assessed, these findings describe systemic biochemical responses; their relationship to skeletal muscle oxidative damage and functional recovery remains to be established.

Uric acid peaked immediately after the LIST and no longer differed significantly from baseline from 24 h onward, although mean values approached baseline only at 72 h, a pattern consistent with its established role as an acute, exercise-intensity-dependent antioxidant response driven by purine nucleotide catabolism and skeletal muscle urate release during high-intensity efforts [28,29]. This immediate rise contrasts with the later, more gradual increase reported for uric acid in some competitive match studies, in which values remained elevated up to 72 h post-match [11]; this discrepancy may reflect differences in absolute exercise intensity, hydration status, or the simulated versus competitive nature of the exercise bout.

Reduced glutathione followed a similarly early rise, peaking at 0 h and remaining elevated at 24 h before returning to baseline by 48 h. This acute increase is consistent with previous reports of changes in the glutathione system immediately following strenuous exercise [30] and with evidence that glutathione is exchanged between skeletal muscle and plasma during exercise [29]. However, the plasma GSH concentration reflects the balance between GSH efflux from tissues and its extracellular degradation, and it may also be affected by exercise-induced shifts in plasma volume. Because only plasma GSH was measured, without oxidized glutathione or direct indices of intracellular redox status, the present data cannot establish whether the increase reflects mobilization of intracellular antioxidant reserves, increased tissue efflux, or reduced extracellular turnover. GGT is a membrane-bound ectoenzyme that initiates the extracellular catabolism of GSH and glutathione conjugates by cleaving their gamma-glutamyl bond, thereby contributing to the recovery of constituent amino acids, notably cysteine, which can be taken up by cells for intracellular GSH synthesis [18,31]. Circulating GGT activity did not change significantly across the recovery period in our sample. Because serum GGT activity largely reflects enzyme released from tissues, mainly the liver, rather than glutathione turnover within skeletal muscle [31], this stability does not allow conclusions about the contribution of glutathione recycling to the plasma GSH changes observed here.

Bilirubin and uric acid both increased after the LIST, and their peak changes were positively correlated in the exploratory analysis. This association is compatible with a concurrent non-enzymatic antioxidant response to exercise, but given the small sample, the number of correlations examined, and the wide confidence interval, it should not be taken as evidence that the two markers share a common regulatory mechanism. This pairing has been reported previously: both markers increase concomitantly following intense exercise [28], and supplementation-based interventions that enhance one non-enzymatic antioxidant have been shown to enhance the other in parallel [32]. Bilirubin’s antioxidant properties are increasingly attributed not only to a passive cytoprotective role but to an active feedback response, whereby oxidative stress upregulates heme oxygenase activity and, consequently, bilirubin production [33,34]. Unlike uric acid, however, bilirubin peaked later, at 24 h, although no pairwise comparison with Baseline reached significance after Holm adjustment, mirroring the delayed kinetics observed for C-reactive protein, and consistent with previous reports that bilirubin and inflammatory markers tend to share a similar delayed time course after soccer match-play [11].

C-reactive protein peaked at 24 h post-LIST, in agreement with the consistently reported 24-h peak of this acute-phase inflammatory marker following football match-play [11,35]. Because C-reactive protein was the only direct inflammatory marker measured, these data describe the systemic acute-phase response rather than the inflammatory response as a whole; other components, such as circulating cytokines or leukocyte counts, may follow different time courses. The exploratory positive correlation between the area under the curve of C-reactive protein and that of uric acid might indicate that players with a larger acute-phase response also showed a larger uric acid response, possibly because both depend on the overall mechanical and metabolic load of the exercise bout. However, this association was not corrected for multiple testing, its confidence interval was wide, and, with 12 participants, it may not be replicated; it should therefore be regarded as a hypothesis for future studies rather than as evidence of a shared physiological mechanism.

The two lipid peroxidation markers diverged sharply in their recovery kinetics. Conjugated dienes, an early product of polyunsaturated fatty acid peroxidation, increased progressively after the LIST and remained significantly elevated at every time point up to 72 h, indicating that this circulating marker of lipid peroxidation had not returned to baseline by the end of the 72-h observation window. MDA, a later and more stable end-product of the same pathway, peaked immediately after the LIST and returned to baseline by 24 h. This divergence is consistent with the distinct kinetics of early versus late lipid peroxidation products described by Vasankari et al. [36] and suggests that plasma conjugated dienes may be a more sensitive circulating marker of lipid peroxidation during the later stages of the post-match period, when MDA has already returned to resting values. Nevertheless, plasma conjugated dienes reflect the net balance between the formation of lipid oxidation products in various tissues, not only skeletal muscle, their transport in circulating lipoproteins, and their clearance from the circulation, and blood and tissue redox biomarkers do not always change in parallel [37]. The persistence of conjugated dienes in plasma therefore cannot be taken as direct evidence that oxidative damage was still ongoing within skeletal muscle at 72 h, which would require tissue-level measurements such as muscle biopsies. The positive exploratory correlation between the peak changes in conjugated dienes and MDA is compatible with both markers reflecting lipid peroxidation, but given its wide confidence interval and the absence of correction for multiple testing, it does not by itself establish that the two markers reflect the same underlying process.

### 4.1. Limitations

This study has several limitations that should be considered when interpreting these findings. First, the sample size was small (*N* = 12), consistent with the highly controlled, laboratory-based, repeated-measures design, but this limits statistical power, increases the risk of both type I and type II errors in the pairwise comparisons, and restricts the generalizability of the correlation analyses, which should be considered exploratory and hypothesis-generating rather than confirmatory. In particular, no a priori power analysis was performed, and the sensitivity analysis (Section 2.4) showed that only large within-participant effects (*dz* ≥ 0.89) and correlations (0.73 or greater in absolute value) could be detected reliably. Given that seven biomarkers were each compared with Baseline at four post-LIST time points, small or moderate changes, for example in GGT or in bilirubin at individual time points, may have gone undetected, and non-significant results should not be interpreted as evidence of no change.

Second, this study did not include a resting, non-exercising control condition. Although the LIST protocol has been extensively validated as reproducing the physiological demands of a soccer match [13], the absence of a control condition means that the contribution of circadian variation or repeated venipuncture to the observed marker fluctuations cannot be fully excluded.

Third, plasma GSH concentrations were measured in isolation, without the concomitant measurement of oxidized glutathione. Because the GSH:GSSG ratio is considered a more sensitive index of the intracellular redox balance than the absolute GSH concentration alone, the present GSH kinetics should be interpreted as a partial, rather than complete, picture of the glutathione antioxidant system’s response to the match.

Fourth, only circulating biomarkers were measured, and C-reactive protein was the only direct marker of inflammation. Oxidative damage within skeletal muscle was not assessed directly. The conclusions of this study are therefore restricted to the plasma biomarkers measured and cannot be extended to tissue-level redox status or to the inflammatory response as a whole.

Fifth, no functional or performance-based recovery measures (e.g., sprint performance, jump height, or maximal voluntary force) and no perceptual indices of recovery were assessed. Consequently, the present data describe the time course of selected circulating biomarkers and cannot indicate whether players’ physical performance had recovered at any time point, nor whether the prolonged elevation of conjugated dienes had functional consequences.

Sixth, dietary intake, hydration status, and daily physical activity during the recovery period were not monitored, and biomarker concentrations were not corrected for exercise-induced changes in plasma volume [38]. Between-participant or between-day differences in nutrition, particularly in the intake of dietary antioxidants, and in hydration status may therefore have contributed to the variability of the circulating markers.

Finally, the findings were obtained from a single simulated match performed by male semi-professional players during the transition from the off-season to the pre-season. Because simulated match-play produces smaller redox disturbances than competitive matches [14], and because training status, sex, competitive level, and the cumulative load of congested schedules may all modify redox and inflammatory responses, the results may not generalize to competitive matches, female or elite players, in-season conditions, or repeated matches within a short period.

### 4.2. Implications for Practice

These findings suggest that, when redox-related biomarkers are monitored after simulated soccer match-play, a single early post-LIST sample, or reliance on C-reactive protein alone, may not capture the full time course of the biochemical response, because C-reactive protein no longer significantly differed from Baseline from 48 h onward, whereas plasma conjugated dienes remained elevated at 72 h. However, the practical significance of this prolonged elevation for performance, injury risk, or readiness to play remains unknown because functional recovery was not assessed, and the present data should not be used on their own to guide return-to-play or selection decisions. Moreover, the present design does not establish that modifying antioxidant intake, or any other recovery strategy targeting lipid peroxidation, would improve recovery. Such strategies should be evaluated in controlled intervention studies, bearing in mind that attenuating exercise-induced oxidative stress may also blunt redox-sensitive training adaptations [6].

## 5. Conclusions

This study showed that a single bout of simulated soccer match-play was followed by significant changes in circulating markers of both lipid peroxidation and antioxidant defense in semi-professional players, with time courses that differed across markers. Uric acid and MDA were significantly elevated only immediately after the LIST; reduced glutathione was affected up to 24 h, and C-reactive protein, the only inflammatory marker assessed, only at 24 h, whereas conjugated dienes remained significantly elevated at every time point up to 72 h. The exploratory correlations observed among some of these markers were not corrected for multiple testing and should be regarded as hypothesis-generating. Overall, among the biomarkers assessed in this study, conjugated dienes showed the most prolonged elevation. Because only circulating biomarkers were measured, these findings indicate prolonged alterations in selected biochemical markers rather than persistent oxidative damage within skeletal muscle or incomplete functional recovery. Studies combining blood-based redox markers with tissue-level and performance measures are needed before such markers can inform recovery monitoring or match-scheduling decisions during congested competitive periods.

## Figures and Tables

**Table 1:** Anthropometric characteristics of the participants (N = 12).

Characteristic	Mean (SD)
Age (years)	23.2 (0.7)
Height (m)	1.81 (0.04)
Body mass (kg)	75.45 (4.69)
Body mass index (kg/m^2^)	22.8 (1.5)
Estimated maximum oxygen uptake (VO_2_max) (mL·kg^−1^·min^−1^)	57.2 (3.7)

**Table 2:** Descriptive statistics (mean [SD] for parametric markers; median [IQR] for non-parametric markers) and omnibus test results for oxidative damage and antioxidant/defense markers across the recovery period following simulated soccer match-play.

Marker	Baseline	0 h	24 h	48 h	72 h	Omnibus Test (Effect Size)
Uric acid (µmol/L)	306.58 (58.58)	367.42 (75.93)	345.00 (51.79)	326.75 (62.77)	302.50 (53.91)	*F*(4, 44) = 8.01, *p* < 0.001, η_p_^2^ = 0.421
Bilirubin (µmol/L)	7.00 (5.75–8.50)	8.00 (8.00–10.50)	11.00 (8.00–12.50)	8.00 (7.75–10.25)	8.00 (6.75–10.00)	χ^2^(4) = 9.99, *p* = 0.041, *W* = 0.208
C-reactive protein (mg/L)	0.80 (0.67–1.70)	0.80 (0.60–1.62)	2.00 (1.30–3.00)	1.20 (0.82–2.40)	1.00 (0.60–1.47)	χ^2^(4) = 20.43, *p* < 0.001, *W* = 0.426
Conjugated dienes (OD units)	0.32 (0.28–0.41)	0.54 (0.44–0.63)	1.26 (1.20–1.31)	1.27 (0.86–1.36)	0.86 (0.75–0.92)	χ^2^(4) = 29.87, *p* < 0.001, *W* = 0.622
Gamma-glutamyl transferase (IU/L)	18.17 (7.00)	17.58 (6.40)	17.08 (7.19)	17.58 (6.27)	16.83 (6.51)	*F*(4, 44) = 1.25, *p* = 0.303, η_p_^2^ = 0.102
Reduced glutathione [GSH] (nmol/mg protein)	13.62 (4.17)	19.76 (6.24)	18.71 (7.11)	14.96 (2.18)	14.65 (2.76)	*F*(2.35, 25.89) = 3.98, *p* = 0.026, η_p_^2^ = 0.266
Malondialdehyde (µmol/L)	5.71 (4.72–6.76)	8.66 (7.82–9.88)	6.71 (4.78–8.14)	7.37 (6.35–8.28)	5.08 (4.25–6.37)	χ^2^(4) = 15.80, *p* = 0.003, *W* = 0.329

**Note.** Repeated-measures ANOVA was used for uric acid, gamma-glutamyl transferase, and reduced glutathione (GSH); the Friedman test was used for bilirubin, C-reactive protein, conjugated dienes, and malondialdehyde. Effect sizes are partial eta-squared (η_p_^2^) for the repeated-measures ANOVA and Kendall’s *W* for the Friedman test. Sphericity was assumed for uric acid (Mauchly’s *p* = 0.052) and gamma-glutamyl transferase (Mauchly’s *p* = 0.366); the Greenhouse–Geisser correction was applied for GSH (ε = 0.59; Mauchly’s *p* = 0.008).

**Table 3:** Pairwise comparisons of each post-LIST time point against Baseline for the markers with a significant omnibus test.

Marker	Comparison	Test Statistic	Raw *p*	Holm-Adjusted *p*	Effect Size
Uric acid (µmol/L)	Baseline vs. 0 h	*t*(11) = −4.57	*p* < 0.001	*p* = 0.003 *	*dz* = 1.32
	Baseline vs. 24 h	*t*(11) = −2.58	*p* = 0.026	*p* = 0.077	*dz* = 0.75
	Baseline vs. 48 h	*t*(11) = −2.13	*p* = 0.056	*p* = 0.113	*dz* = 0.62
	Baseline vs. 72 h	*t*(11) = 0.36	*p* = 0.729	*p* = 0.729	*dz* = −0.10
Bilirubin (µmol/L)	Baseline vs. 0 h	*W* = 0.0	*p* = 0.031	*p* = 0.125	*r* = 0.45
	Baseline vs. 24 h	*W* = 7.0	*p* = 0.037	*p* = 0.125	*r* = 0.43
	Baseline vs. 48 h	*W* = 18.0	*p* = 0.208	*p* = 0.416	*r* = 0.27
	Baseline vs. 72 h	*W* = 14.5	*p* = 0.688	*p* = 0.688	*r* = 0.10
C-reactive protein (mg/L)	Baseline vs. 0 h	*W* = 0.0	*p* = 0.062	*p* = 0.188	*r* = 0.42
	Baseline vs. 24 h	*W* = 2.0	*p* = 0.006	*p* = 0.023 *	*r* = 0.53
	Baseline vs. 48 h	*W* = 11.0	*p* = 0.102	*p* = 0.203	*r* = 0.34
	Baseline vs. 72 h	*W* = 27.0	*p* = 0.980	*p* = 0.980	*r* = 0.01
Conjugated dienes (OD units)	Baseline vs. 0 h	*W* = 0.0	*p* < 0.001	*p* = 0.002 *	*r* = 0.62
	Baseline vs. 24 h	*W* = 1.0	*p* < 0.001	*p* = 0.002 *	*r* = 0.61
	Baseline vs. 48 h	*W* = 0.0	*p* < 0.001	*p* = 0.002 *	*r* = 0.62
	Baseline vs. 72 h	*W* = 1.0	*p* < 0.001	*p* = 0.002 *	*r* = 0.61
Reduced glutathione [GSH] (nmol/mg protein)	Baseline vs. 0 h	*t*(11) = −4.43	*p* = 0.001	*p* = 0.004 *	*dz* = 1.28
	Baseline vs. 24 h	*t*(11) = −3.05	*p* = 0.011	*p* = 0.033 *	*dz* = 0.88
	Baseline vs. 48 h	*t*(11) = −0.98	*p* = 0.347	*p* = 0.694	*dz* = 0.28
	Baseline vs. 72 h	*t*(11) = −0.66	*p* = 0.523	*p* = 0.694	*dz* = 0.19
Malondialdehyde (µmol/L)	Baseline vs. 0 h	*W* = 0.0	*p* < 0.001	*p* = 0.002 *	*r* = 0.62
	Baseline vs. 24 h	*W* = 23.0	*p* = 0.233	*p* = 0.467	*r* = 0.26
	Baseline vs. 48 h	*W* = 11.0	*p* = 0.027	*p* = 0.081	*r* = 0.45
	Baseline vs. 72 h	*W* = 34.0	*p* = 0.733	*p* = 0.733	*r* = 0.08

**Note.** Paired-samples *t* tests (with Cohen’s *dz*) were used for the repeated-measures ANOVA markers; Wilcoxon signed-rank tests (with matched rank-biserial “*r*”) were used for the Friedman-test markers. “*p*” values are Holm-adjusted within each marker. Asterisks (*) denote Holm-adjusted *p* < 0.05. Pairwise comparisons were not performed for gamma-glutamyl transferase because its omnibus test was not significant (Table 2).

**Table 4:** Exploratory Spearman correlations between oxidative stress markers.

Marker Pair	Basis	Spearman *r* [95% CI]	*p*
Conjugated dienes vs. Malondialdehyde	Peak change	*r* = 0.60 [0.04, 0.87]	*p* = 0.039
Uric acid vs. Bilirubin	Peak change	*r* = 0.65 [0.12, 0.89]	*p* = 0.023
Uric acid vs. C-reactive protein	AUC (post-LIST)	*r* = 0.71 [0.23, 0.91]	*p* = 0.009

**Note.** Only nominally significant correlations (uncorrected *p* < 0.05) are reported. No correction for multiple testing was applied, and none of these correlations remained significant after Holm correction for the 42 tests was performed. Correlations were computed using each marker’s peak change from Baseline (Baseline to peak value) across participants, except where noted as based on the area under the curve (AUC) across the post-LIST time points (0 h to 72 h). CI, confidence interval (Fisher’s *z* transformation).

## Data Availability

The data presented in this study are available on request from the corresponding author.

## Abbreviations

The following abbreviations are used in this manuscript:

ANOVA	Analysis of variance
AUC	Area under the curve
BIL	Bilirubin
CI	Confidence interval
CRP	C-reactive protein
DTNB	5,5′-dithiobis-(2-nitrobenzoic acid)
EDTA	Ethylenediaminetetraacetic acid
GGT	Gamma-glutamyl transferase
GSH	Reduced glutathione
GSSG	Oxidized glutathione
IQR	Interquartile range
LIST	Loughborough Intermittent Shuttle Test
MDA	Malondialdehyde
OD	Optical density
ROS	Reactive oxygen species
SD	Standard deviation
UA	Uric acid
VO_2_max	Maximal oxygen uptake
